# Small Animal Models to Study Herpes Simplex Virus Infections

**DOI:** 10.3390/v16071037

**Published:** 2024-06-27

**Authors:** Mohammed Tanveer Hussain, Brent A. Stanfield, David I. Bernstein

**Affiliations:** 1 Division of Biotechnology and Molecular Medicine and Department of Pathobiological Sciences, School of Veterinary Medicine, Louisiana State University, Baton Rouge, LA 70803, USA; 2 Cincinnati Children’s Hospital Medical Center, University of Cincinnati, Cincinnati, OH 45229, USA

**Keywords:** human herpesviruses, herpes simplex virus (HSV), animal models, mouse models, rabbit models, guinea pig models

## Abstract

Herpes simplex virus type 1 (HSV-1) and herpes simplex virus type 2 (HSV-2) are two of the most prevalent human viruses worldwide. They are known to cause a variety of diseases including genital herpes, meningitis, encephalitis, cold sores and herpes stromal keratitis. The seropositive rate for HSV-1 is around 90%, whereas for HSV-2 it remains around 20–25% for the general adult population. The infections caused by these viruses remain difficult to study because a large proportion of infected individuals are asymptomatic. Furthermore, given the neurotropic characteristics of the virus, studies aimed at understanding the complex pathogenesis in humans is difficult. As a result, animal models have been developed to understand several characteristics of HSV biology, pathogenesis, disease and host responses to infection. These models are also commonly used as the first evaluation of new drugs and vaccines. There are several well-established animal models to study infection with HSV, including mice, guinea pigs and rabbits. Variables within the animal models depend on the species of animal, route of infection, viral strain, dosage, etc. This review aims at summarizing the most commonly used animal models to study HSV pathogenesis and therapies.

## 1. Introduction

Human herpesviruses are large, double-stranded DNA viruses, which have a high disease prevalence around the world [1]. They belong to the family of Herpesviridae and can be categorized into subfamilies of alpha, beta or gamma herpesvirinae. The different viruses are grouped based on shared characteristics such as cellular tropism, replication dynamics and genomic organization. The ability to cause severe infections, combined with limited treatment options, makes herpesviruses a significant public health concern [2]. Herpes simplex viruses are grouped within the alphaherpesvirinae family, and there are two serotypes of the virus: herpes simplex type 1 (HSV-1) and herpes simplex type 2 (HSV-2). HSV-1 is more commonly associated with ocular and oral mucosal infections, whereas HSV-2 is primarily linked to genital tract infections [3]. Around the world, an estimated 3.7 billion people under the age of 50 (67%) are infected with HSV-1, whereas 491 million people aged 15–49 (13%) suffer from HSV-2 infections [4].

Within healthy individuals, HSV infections are often asymptomatic or cause mild and self-limiting disease. In the immunocompromised, including neonates, HSV infection can be associated with high mortality and morbidity [5]. The active replication of HSV-1 and HSV-2 can lead to diseases such as herpes stomatitis, genital herpes, herpes stromal keratitis, eczema herpeticum, encephalitis and others. The ability of HSV to persist in sensory neurons and periodically reactivate for the lifetime of the infected host adds significantly to its public health burden [6]. The interaction with HIV that enhances infection by both viruses has also added greatly to the interest in developing strategies to combat HSV [7].

Apart from in vitro infection models that utilize primary and immortalized human cell lines [8,9,10], advanced coculture, tissue culture and “organoid” systems have been utilized to study the dynamics of herpesvirus infections [11,12,13,14]. These systems provide useful information to investigate the basic principles of viral–cellular interactions. However, they remain inadequate given that development of successful antiviral drugs and vaccines require an in-depth understanding of infection dynamics and host immune responses on a systemic level. 

Animal models are crucial because they allow researchers to develop a deep understanding of complex biological systems that underlie the host’s ability to combat pathogens. Furthermore, scientific studies in animal models can help mitigate the many variables including environmental, strain, dose and inoculum size existing in clinical research [15]. Recently, the need for understanding HSV infection on a systematic level has led to the use of various animal models. Small animals such as mice, rabbits and guinea pigs are frequently employed to study HSV infections, while less commonly used animals include tree shrews, zebrafish and cotton rats [16,17,18,19,20,21]. In this review, we discuss the various nonprimate animal models utilized to study HSV infections and briefly summarize the animal models including characteristics such as disease development, route of infection and metrics of disease. Subsequently, we highlight the utility of these models to investigate herpes simplex virus infections and interventions in recent years. 

## 2. Mouse Models for Studying Human Herpes Simplex Virus Infections

In humans, HSV primarily infects the epithelial cells, fibroblasts and keratinocytes during acute replication. Cellular entry is primarily mediated by binding of the viral glycoproteins (B and C) to the surface receptors called herpesvirus entry mediator (HVEM), nectin-1 and nectin-2, resulting in distinct entry mechanisms of fusion between the cellular plasma membrane or endocytosis [22,23,24] In mice, several of the entry receptors used by HSV are similar enough to humans to permit entry, enabling HSV-1 and HSV-2 to successfully infect mice. This enables the utilization of mice as an interspecies infection model for HSV. Murine epithelial cells and fibroblasts both effectively express HVEM and nectin-1, and this has enabled the study of HSV-1 infections within various mouse tissues such as the eye, mucosal membranes and skin [25,26].

**Figure 1:**
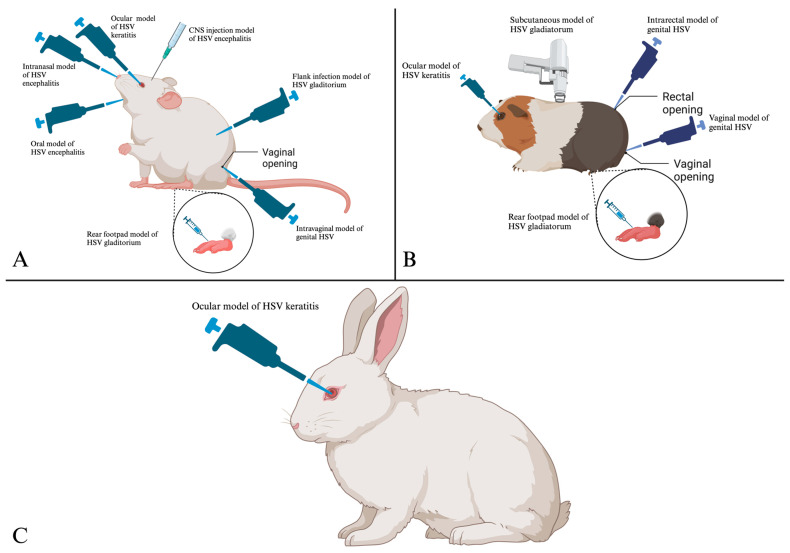
Routes of infection and establishment of HSV disease within mice (**A**), guinea pigs (**B**) and rabbits (**C**). Created with Biorender.com.

Mice have been used to study ocular HSV, oral HSV, HSV encephalitis and genital HSV. To study the onset of ocular herpes infections, a low dose of HSV-1 is usually applied to a small scratch within the cornea of the eye, resulting in a localized infection (Figure 1). Progression is monitored by gross examination of pathology and through the analysis of infectious virus in swab samples to quantify the amount of virus shedding from the infected eye [27] (Table 1). Shimeld et al. were among the first to pioneer the use of mice to study recurrent herpetic eye diseases [28]. Others have developed methods to induce reactivation, allowing for the study of recurrent disease [29,30]. In recent years, the ocular model has been used to study various therapeutic approaches to mitigate herpes stromal keratitis, including the use of CRISPR to reduce the latent viral load in infected animals [31,32].

Mice have been extensively used to study oral mucosal infections caused by HSV-1 by inoculation into the oral tissues, which closely resembles the natural route of human exposure (Figure 1). The infection can be established through injection of a viral solution into the lip or tooth pulp or through topical application of the virus to the lip muco-epithelium (Table 1). The progress of disease can be measured through monitoring loss of body weight and lip lesions [33,34]. The lip infection model most notably has been utilized to study a trivalent modified mRNA vaccine which has been shown to protect mice from both genital and non-genital HSV-1 infections [56].

Furthermore, intranasal inoculations (IN) with HSV-1 and HSV-2 have widely been used to study the onset of encephalitis and related immune responses. Following local replication, the virus infects the brain, causing a lethal encephalitis (Figure 1). The IN infection model is widely utilized to study potential therapeutics to combat HSV (Table 1) [35,36]. Uhlig et al. utilized this infection model to show that helicase primase inhibitors are potential HSV therapeutics for combatting oral mucosal infections [57]. Additionally, the role of stress and lipid peroxidase in susceptibility to HSV infections has been uncovered utilizing the IN model [58].

The productive infection of HSV in the skin requires compromising the skin integrity given that HSV cannot penetrate the keratinized epidermis. Mice have been commonly used to study cutaneous and subcutaneous HSV-1 infections, with the rear footpad and flank being the most common inoculation sites (Table 1) [37]. Clinical manifestations such as tremors, weight loss and inflammation at the site of injection are utilized to monitor the progression of the disease [59,60]. The flank infection model has recently been used to show that the recruitment of γδT cells to the site of infection leads to enhanced pathogenesis of subcutaneous HSV infections [61]. This contrasts with reports regarding the role of γδT cells in the protection of ocular tissue induced from vaccination [62]. Injection into the rear footpad represents another model for studying subcutaneous HSV infections, which results in a reproducible infection and signs of disease including hair loss, edema, paralysis and necrosis of the foot [38]. The model can also be used to study HSV-induced encephalitis when neurovirulent strains of the virus are used to infect immunodeficient mice. Laval et al. adapted this model to study the initiation and development of neuroinflammatory responses during herpesvirus infections using the closely related pseudorabies virus [63]. The infection is found to spread from the primary site of infection to sensory and sympathetic nerve fibers found within the skin before travelling to the sciatic nerve and the dorsal root ganglia, eventually making its way to the brain via the spinal cord [64]. Asanuma et al. adapted the footpad infection model to study the effects of herpes simplex virus infections within sweat glands to demonstrate how HSV infection affects sweating and infection-induced dry skin [65]. The footpad infection model has also been utilized to study the role of virally encoded microRNAs (miR) miR-H1 and miR-H6 in reactivation of HSV-1 from latently infected dorsal root ganglia ex vivo [66].

The ability of HSV to infect the nervous system can also be studied by directly injecting HSV into the central nervous system (CNS). This approach is useful for studying the role of a particular gene and how it affects HSV infection within the brain [49]. The virus can be injected into various areas of the brain including the olfactory bulb, hippocampus, sinus confluences and lateral ventricle (Figure 1) [39,40]. Following inoculation, inflammatory responses limit viral replication and initiate immune responses to control viral spread. The cellular responses of brain cell populations can be studied by removing the cells from the study animal, followed by purification and infection in vitro. In recent years, the infection model has been utilized to study the role of host genes in HSV encephalitis (HSE). Zeng et al. utilized the model to show that β-Arrestin-2 is highly expressed in brain tissues of HSE mice and overexpression leads to protection against neurological degradation [67]. β-Arrestin-2 overexpression has been associated with the formation of Aβ plaques formed in Alzheimer’s disease patients [68]. In contrast to the findings from Zeng et al., Bocharova et al. demonstrated that Aβ plaques formed during Alzheimer’s disease do not exhibit a protective effect against HSV; it was also shown that infection with HSV does not induce the formation of Aβ plaques [69]. The model has also been utilized to study various therapeutics, including anti-inflammatory effects of chemicals on HSV-1-induced encephalitis [70]. Direct infection of the murine CNS offers a unique model system to probe HSE and the effect of HSV infection in neuronal pathologies including Alzheimer’s disease.

Genital HSV infections in mice result in severe acute disease typically resulting in hind limb paralysis and death within the first 8 days of infection [71]. To study genital herpes, female mice are infected vaginally with either HSV-1 or HSV-2 (Figure 1) following pretreatment with medroxyprogesterone to synchronize the estrous cycles and cause a thinning of the uterine lining [41,72]. The inoculation causes local genital lesions, and progress can be monitored through lesion assessment, body weight measurement and development of hind limb paralysis followed by animal death at day 6–8 postinfection [73]. In recent years, the genital herpes model has been utilized to test the efficacy of several vaccine candidates. Görander et al. utilized the model to test the efficiency of a truncated glycoprotein-G vaccine against genital HSV-2 challenge, wherein protective effects were found in vaccinated mice, and this protection was associated with non-neutralizing antibody responses [74]. Additionally, the model has been widely utilized to uncover the functions of various genes and test the efficacy of several antiviral therapeutics [63,75,76,77,78]. The mouse model of genital HSV infection offers an efficient small animal model to evaluate therapeutic efficacy of interventions targeting acute disease, but because this is a lethal model, it cannot be used to study recurrent disease.

## 3. Guinea Pig Models for Studying Herpes Simplex Virus Infections

Guinea pigs are widely accepted as the gold standard animal model to study genital herpes simplex virus infections and spontaneous viral reactivation [79]. The model has also been used widely to study the efficacy of HSV vaccines and antivirals due to its ability to closely mimic the acute genital tract infection and recurrent ulcerative disease observed in humans [80]. Recurrent lesions can be observed spontaneously for at least 2 months following resolution of acute disease and induced through exposure of latently infected animals to ultraviolet light or anti-inflammatory steroids. Animals also exhibit recurrent virus shedding even in the absence of lesions as seen in humans. Thus, infection in guinea pigs mimics the key elements of human disease, allowing the evaluation of drugs and therapeutic vaccines for recurrent HSV infections which cannot be studied within mouse models [79]. 

To study genital herpes simplex virus infections, female guinea pigs are challenged through intravaginal inoculation without modifying the integrity of the vaginal mucosa or using progesterone (Figure 1) [81]. Following intravaginal inoculation, the virus replicates in the genital mucosa and can be quantified by obtaining vaginal swabs. The highest viral titers can be found within the introitus, vagina and bladder, which are considered the primary sites of acute viral replication. From these sites, the virus enters into the axonal termini of sensory neurons within one or two days postinfection [82]. The virus then traffics to the dorsal root ganglia and spinal cord to establish a “latent” infection.

After latency is established, the virus in the dorsal root ganglia and spinal cord can reactivate to induce lytic replication after which the infectious virus travels through the peripheral nerves into the external genital skin, where it can produce recurrent lesions within the genital area or genital virus shedding in the absence of lesions [79]. The extent of primary genital disease can be quantified by observing the genital area for lesions and assigning a score based on the severity of the lesions. For acute disease, lesions are scored until 11–14 days postinfection, whereas recurrent disease is commonly measured from 15 to 70 days postinfection [42]. Recurrent disease usually manifests as a single lesion which rapidly heals.

In recent years, the guinea pig model has been utilized to study several notable vaccine candidates as both prophylactic and therapeutic vaccines. Prophylactic vaccine studies designed to prevent or limit acute disease and the establishment of latency included protein vaccines with several adjuvants [83], DNA vaccines [84], mRNA vaccines [85] and live attenuated vaccines [86]. For example, we utilized this model to study the efficacy of an inactivated HSV-2 vaccine in conjugation with an adjuvant to show that protective effects were induced against heterologous and homologous HSV strains [87,88]. Egan et al. tested the efficacy of a trivalent protein vaccine containing glycoproteins C, D and E to show that protection was induced against HSV-1 genital infections [83]. Likewise, we utilized the genital infection model to test the efficacy of the HSV-1 VC2 vaccine which contains mutations within the viral glycoprotein-K and UL-20. The study demonstrated the efficacy of the vaccine, with decreased clinical severity and recurrent HSV-2 disease within the animals following prophylactic vaccination [86]. Furthermore, Awasthi et al. utilized the model to show that an mRNA vaccine encoding viral glycoproteins induced more protective efficiency as compared to administration of the glycoproteins alone, leading the way for new mRNA vaccines against HSV [85]. Similar strategies have been used for therapeutic vaccines designed to prevent or limit recurrent lesions and recurrent virus shedding. The prime-and-pull vaccination strategy relies on two steps, where the first step utilizes a conventional vaccination to elicit systemic T-cell responses (prime) followed by a topical administration of T-cell attractants (pull) to establish localized long-term protective immunity [88]. The evaluation of a prime-and-pull strategy for therapeutic vaccines showed the requirement for both the vaccine prime and a TLR 7 agonist pull for optimal effectiveness [88]. Current evaluations of very potent mRNA vaccines are also underway, although the optimum antigens to be included have not been defined. The guinea pig model of genital herpes infection remains the best model of human disease for these preclinical intervention studies [63,86,89,90,91,92,93].

Alternatively, an intrarectal model to study genital herpes in males was developed by Bourne et al. (Table 1) [43]. The process involves inoculation of the virus within the rectal cavity of the guinea pigs, causing primary genital ulcerative disease and recurrent disease observed within 15–50 days postinfection. The progress of disease can be monitored through daily observation for ulcerative lesions, while virus replication can be quantified by collecting rectal swabs to assess recurrent viral shedding. 

Although not commonly used, previous studies have utilized a cutaneous model to study herpes simplex virus infection with guinea pigs [44]. In this model, the backs of guinea pigs are depilated, and percutaneous inoculation is administered using a vaccination gun. Lesions develop at the site of inoculation, and progress can be monitored by measuring the size of the lesions and collecting skin for virus titrations [94]. Another skin infection model is the rear footpad infection model, as described by Iwasaka et al. [45], where they describe the inoculation of the virus into the rear footpad, with clinical symptoms appearing 24–48 h postinfection. The virus shedding can be detected 5–8 days postinfection, and a large percentage of animals developed recurrent disease [45].

Apart from skin and genital infections, Yadavalli et al. developed a guinea pig model to study ocular infections of HSV [46]. During the process, the guinea pig corneas were scarified and the virus was applied to the eye surface, much akin to the mouse model. The progress of disease was then monitored through symptoms of disease such as weight loss, paralysis, seizures, encephalitis, etc. The viral titers were performed to quantitate the amount of virus present within the infection site, and the corneal opacity score was assigned to monitor the progress of ocular disease. The model was also shown to be useful in studying recurrent herpes disease given that spontaneous reoccurrence was observed within several animals.

## 4. Rabbit Models for Studying Herpes Simplex Virus Infections

Rabbits have widely been used in biomedical research due to their size and phylogenetic relatedness to primates [95]. For HSV, rabbits have been utilized to study ocular HSV-1 infections as they tend to have large eyes, making examination of corneal lesions readily accessible for imaging and quantification (Figure 1). Furthermore, similarity to human disease and spontaneous reactivation within rabbits infected with high phenotypic reactivator (HPR) strains of HSV-1 render them a suitable model for studying HSV. The relatively large size of rabbits also provides more infective tissue for assessment, and tear films allow for more tear collection [47]. However, in comparison to other small animals such as mice and guinea pigs, utilizing rabbits is expensive and requires special husbandry needs. The most commonly used rabbit strains to study ocular herpes include New Zealand white (NZW) and Dutch belted rabbits. The NZW is most commonly used because it has nonpigmented eyes, making it particularly useful for ocular research. 

For the model, HSV-1 is inoculated following mild scarification of the cornea in a grid pattern using a sterile needle or abrasion using cotton tipped applicators. Following infection, the tears of the rabbits can be collected daily, and infectious virus titers can be quantified using a standard plaque assay. The eyes can be observed using a slit-lamp microscope every day postinfection, and the severity of the conjunctivitis is assessed using a scoring method for six clinical parameters, as described by others [47]. In recent years, the ocular model has been widely used to understand the genetic mechanisms behind herpes ocular infections. Washington et al. utilized the model to show that HSV reactivation from latency is driven through depletion of the CTCF insulator protein [96]. Similarly, Singh et al. utilized the model to uncover the functions of the CTRL2 insulator protein, as deletion was shown to result in reduced translation of genes driving axonal transport and attenuation of reactivation from latency [97]. Barozzo et al. showed that deletion of the miR-H8 miRNA has no effect on HSV-1 viral loads during reactivation and is dispensable during establishment of latency [98], while the microRNAs miR-H1 and miR-H6 are required for efficient reactivation of HSV-1 from latency [66]. The model has also been utilized to study the efficacy of various antiviral therapies [99,100].

## 5. Other Models

### 5.1. Transgenic Rabbits

A major disadvantage of using animal models is the differences between the animals’ immune response and human immune responses. HLA-transgenic mice have proven to be useful to study T-cell responses to human epitopes following ocular infections [101,102]. However, herpetic conjunctivitis differs in mice compared to humans and rabbits [48]. Therefore, transgenic rabbit models are useful tools in studying humanized immune responses to HSV infections, further offering the advantage of studying HSV reactivation. HLA-A*0201 transgenic rabbits have been generated to study humanized immune responses to HSV [48]. In this model, the eyes of the transgenic rabbits are inoculated with the virus, and shedding is quantified by measuring viral titers within the tears of infected animals. The humanized immune responses can then be monitored. For example, Chentoufi et al. [48] utilized the model to show that subcutaneous injection of the rabbits with CD4 and CD8 epitope-based HSV-1 gD lipopeptides resulted in induction of CD4^+^ and CD8^+^ T-cell responses. Vaccination also decreased HSV-1 replication in the tears and reduced ocular disease following HSV challenge [101]. Another publication from the same research group utilized the model to show that HSV-1 gD epitopes derived from HSV-1 seropositive asymptomatic healthy individuals resulted in adequate HSV-specific CD8^+^ T-cell activation and significantly reduced HSV-1 shedding in tears [103]. Additionally, the transgenic rabbit model has helped broaden our understanding of HSV immune responses and therapeutics [91,104].

### 5.2. Neonatal Models

Neonatal infections caused by the herpes simplex virus affect around 1/3200 live births in the US, and greater than half of untreated infants develop disseminated disease and encephalitis. Even in the face of antiviral treatment, a mortality of 29% for disseminated neonatal HSV disease and 4% for CNS HSV is seen [105]. The neonatal mice model has provided a unique solution to understanding neonatal HSV infections. In this model, young mice, usually around 7 days old, are inoculated with the HSV viruses, given that 7-day-old mice closely resemble the immunologic maturity of humans at birth [106]. The mice are primarily infected using three different routes. Intraperitoneal, intranasal or intracranial inoculation is most commonly used in neonatal studies (Table 1). Following inoculation, the mice are monitored for signs and symptoms of disease and euthanized under strict humane endpoint criteria. Following euthanasia, the mice organs are harvested and evaluated for the amount of virus present [49]. The cells and organs may also be used for a variety of downstream applications such as immunohistochemistry, ELISA, qPCR, etc. The model was used to show that maternal immunization elicited protective effects to the neonates and played a role in preventing neonatal HSV infection [107,108]. In recent years, the neonatal mouse model has been used to study a wide range of vaccine candidates. Patel et al. utilized the model to test the efficacy of a trivalent glycoprotein vaccine containing gC2, gD2 and gE2 to mediate HSV pathogenesis. Maternal immunization with the vaccine was shown to protect the offspring from neonatal HSV-2 dissemination and disease [109]. Similarly, LaTourette II et al. utilized the model to show that an mRNA vaccine encoding the same glycoproteins was effective in protecting offspring of immunized mothers against HSV-2 challenge [110]. Additionally, studies suggest that neonatal protection is mediated by passive transfer of maternal antibodies to the neonates [111].

Similarly, the neonatal guinea pig model has also been utilized to study neonatal herpes simplex virus infections [50]. The model requires infection of newly born guinea pigs within 48 h of birth. HSV-2 challenge can be applied intranasally or via the cutaneous route; the former requires direct inoculation of the virus within the nasal cavity, whereas for the cutaneous route the scalp must be depilated, scarified and inoculated with the virus. Following inoculation, animals develop clinical manifestations of HSV disease including skin lesions, ocular changes, seizures and weight loss. Recurrent herpes lesions can also be observed in animals that successfully recover from initial HSV-2 infections. In animals that become moribund, humane sacrifice and downstream applications of the organs and tissues prove useful for further analysis. In recent years, the neonatal guinea pig model has been utilized to study the effects of antibody administration into HSV-infected neonatal guinea pigs alongside acyclovir. The data from the study indicated that the addition of antibody therapy alongside conventional therapeutics resulted in improved outcomes of neonatal HSV [112]. Likewise, the model has been utilized to show that N-methanocarbathymidine ((N)-MCT) is more efficient in the treatment of neonatal HSV in comparison with acyclovir [113]. The model has also been utilized to study the effects of age and route on the outcome of neonatal HSV [114].

### 5.3. Cotton Rats

The cotton rat is a well-established laboratory animal and is used to study several diseases, including measles, parainfluenza virus, etc. Lewandowski et al. were the first group to utilize cotton rats to study herpes simplex virus infections. In this model, the lips of cottons rats are scratched and HSV-1 is swabbed onto the area (Table 1). Disease progression is monitored through formation of lesions until 10 days postinfection. The rats can then be euthanized, and the viral titers are measured within the brainstem, cerebelli and trigeminal ganglia. The tissues are also utilized for immunohistochemical detection of HSV-1 antigens within the infected tissues [51]. 

The same group also developed a cotton rat model to study genital herpes [52]. The model utilized inoculation of the virus into the vaginal cavity of medroxyprogesterone-treated female cotton rats. The progression of disease leads to genital lesions, and viral shedding can be detected through collecting swabs and titration of the viral loads through plaque assay. In recent years, the cotton rat model has been utilized to study the effects of HSV-2 glycoprotein-D vaccination against genital HSV-2 and HSV-1 infection. Boukhvalova et al. found that vaccination with the HSV-2 gD vaccine resulted in protection of cotton rats against HSV-1 infections but not HSV-2 genital disease [20], mimicking the results of the large human trial [115]. The same group utilized the model to show that HSV-1 infection within cotton rats leads to demyelination of the central nervous system and may lead to disorders caused due to damage of the central nervous system [116]. Eide et al. utilized the cotton rat model to test the efficacy of peptide-conjugated morpholino oligomers (PPMOs) that inhibit translation of mRNA, leading to decreased protein synthesis. The PPMOs were targeted against ICP0, ICP27, UL-30 and UL-39, and administration led to a reduction of genital lesions and vial shedding within the treated animals [117].

### 5.4. Tree Shrew

The tree shrew is a small animal that is found in southwest Asia, and recent genomic analysis suggests that it is closely related to primates [118]. Furthermore, it can be infected with HSV-1 and HSV-2, making it a worthwhile addition to the repertoire of animal models used to study HSV pathogenesis as an alternate to primates [119]. A tree shrew model of ocular HSV-1 infections was first described by Li et al. (Table 1). Following scarification of the eye and infection through virus inoculation, the animals developed symptoms of disease such as weight loss, ruffled fur and lack of movement [53]. Around 10% of the animals exhibited neurological symptoms similar to human encephalitis followed by death within two weeks. In the animals that survived challenge, the virus could be detected within the trigeminal ganglia 4 weeks postinfection, and LAT intron signals could be detected within the trigeminal ganglia 2 months postinfection, demonstrating that latency can be established and persists in the model. The analysis of tree shrew tears revealed that they continue to shed virus spontaneously at low frequencies. Gu et al. utilized the tree shrew model of HSV to show that stimulation of interferon genes (STING), in particular tGBP1, combines with tSTING to promote autophagy and moderately inhibit HSV-1 infection and spread [120]. Additionally, the tree shrew has proved useful in characterizing DNA damage and transcriptome analysis following HSV-1 infections [121,122].

### 5.5. Zebrafish

Zebrafish are found to naturally express the receptors for HSV binding and entry [123], which are required for their embryonic development and these are the basis for mediating HSV-1 infection of zebrafish [124]. Experimental infection of zebrafish can be achieved through a variety of routes (Table 1). Firstly, it is possible to infect zebrafish by incubating them within an E3 medium containing the virus. Use of a *Lac* Z reporter system allows for quantification of the infected fish through the X-gal assay [54]. Similarly, the fish can be grown in a medium containing fluorescently tagged virus particles that can be tracked using fluorescent microscopy. These models have been useful to study viral tropism, spread and growth kinetics [125] and can also be used for studying inflammation and viral spread from the site of infection [126]. Another widely used technique to infect zebrafish is through intraperitoneal injections in anesthetized fish [55], whereas an alternate route includes inoculation of virus within wounds created by scraping, which facilitates a local infection. In recent years, the zebrafish model has been utilized to study viral tropism. Burgos et al. demonstrated that microinjection of the virus within abdominal cavities of zebrafish resulted in spread of the virus to the brain [55]. Additionally, the immune system of the zebrafish resembles the human immune system, as innate and adaptive immunity develops 4 days postfertilization [127] including Toll-like receptor molecules, homologues of complement and cytokine genes [128,129]. The quick breeding time and low maintenance costs of zebrafish provide useful advantages over traditional animal models of HSV and may serve as a useful foundation to better understand HSV infection dynamics and host response.

## 6. Conclusions

The use of animal models to study infection and disease remains an invaluable tool to help understand herpes simplex virus pathogenesis as well as for the preclinical evaluation of vaccines and antivirals. The selection of an appropriate animal model requires an understanding of the limitations of the model in mimicking human primary and recurrent disease. Unfortunately, infections within animals are never identical to disease in humans, but studies using animal models have furthered our understanding of human disease and serve as an important preclinical step for the evaluation of therapeutics. We presented several animal models available that allow the study of HSV-1 and HSV-2 infections (Table 1). These models remain crucial in understanding infection, latency, reactivation and recurrent disease. Given that different animal models use various infection routes and metrics of disease, the interpretation of the results must be conducted carefully. 

There are many studies that utilize the animal models discussed in this review, and we hope that evaluations using these models helps in the development of more effective treatments and an effective vaccine. Studies that aim to reduce the incidence of recurrent disease and recurrent shedding are critical to reducing the spread of this common infection. With respect to the particular animal models, the mouse models of HSV pathogenesis remain the most commonly utilized given their wide availability and economical advantage. The use of the mouse model has helped shape our understanding of HSV disease, and in recent years, genetically modified mice have helped define functions of HSV gene and gene products. They are also extensively used for the initial testing of vaccine candidates. Similarly, the knowledge of HSV infections gained from studies within rats have proved useful given the known similarities between the human and rat nervous systems. The guinea pig models have been widely utilized to understand the neurotropic characteristics of HSV and remain the most commonly used model to study recurrent disease and therapeutic vaccines. The neonatal models have been used to examine new therapies for this devastating disease, while the rabbit models have been at the forefront to study recurrent ocular HSV. The tree shrew model for studying HSV-1 infections helps gain an understanding regarding HSV-1 infections given their similarities to human anatomy, neurodevelopment and immune responses. Furthermore, unlike mice in which HSV-1 does not spontaneously reactivate, tree shrews experience reactivation and recurrent mucosal lesions. The relative cost and lack of wide availability of tree shrews limits the use of the animal to only a few groups. 

However, the translation of successful animal vaccine studies into humans has been challenging for several reasons. While animal models provide valuable insights, the human immune system is complex, and responses to a vaccine candidate may be different compared to any animal, leading to discrepancies between results in animal models and human trials. This may in part be due to the fact that HSV has evolved and adapted to humans over a very long time, establishing immune evasion strategies that may not be present in animals. It is also important to understand the differences in the design of trials used in clinical vaccine evaluations compared to those used in animal models. For example, animal trials are far smaller than human efficacy trials and are conducted over a much shorter time span and may use different definitions of efficacy [89].

Thus, the goal of accurately replicating every aspect of the immune response and clinical disease within animal models remains difficult. Therefore, the selection of an animal model should depend on a focused question, and the model system should be chosen to best answer that question, keeping in mind the cost and effort. Although there may never be an animal model that precisely mimics all aspects of human disease and immune responses, these models continue to serve as crucial tools in understanding HSV disease and as a screening tool to evaluate new approaches to treating and preventing HSV infections prior to human trials.

## Figures and Tables

**Table 1:** Summary of small animal models used for studying herpes simplex virus.

Model Organism	Route of Inoculation	Virus Strain Used	Refs.
Mice	Ocular	HSV-1 (17 Syn^+^, LAT^−^, LAT^+^)	[27]
	Oral	HSV-1 (H129), HSV-1 (17 Syn^+^)	[33,34]
	Intranasal	HSV-1/F, HSV-1 (strain ID 2762)	[35,36]
	Flank	HSV-1 strain NS	[37]
	Rear footpad	HSV-1 (multiple strains)	[38]
	Injection into CNS	HSV-1 strain SC16, HSV-1 (McKrae, McKrae GFP, tomato red)	[39,40]
	Intravaginal	HSV-2	[41]
Guinea Pigs	Intravaginal	HSV-2	[42]
	Intrarectal	HSV-2 (strain MS)	[43]
	Cutaneous	HSV-1	[44]
	Rear footpad	HSV-2 (strain G)	[45]
	Ocular	HSV-1 (McKrae)	[46]
Rabbits	Ocular	HSV-1 (multiple strains)	[47]
Transgenic Rabbits	Ocular	HSV-1 (McKrae)	[48]
Neonatal Mice	Intraperitoneal	HSV-2 Delta/7–15	[49]
	Intranasal		
	Intracranial		
Neonatal Guinea Pigs	Intranasal	HSV-2, MS strain (ATCC VR-540)	[50]
	Cutaneous		
Cotton Rats	Oral	HSV-1 strain F	[51]
	Intravaginal	HSV-2 (strain G)	[52]
Tree Shrew	Ocular	HSV-1 (17 Syn^+^, McKrae)	[53]
Zebrafish	Incubation	HSV-1(KOS) tk12, HSV-1(KOS) gL86, HSV-1(K26GFP)	[54]
	Intraperitoneal	HSV-1(KOS)	[55]
